# A High-Risk Rigid Bronchoscopy for a Patient With a Mediastinal Mass Causing Superior Vena Cava Syndrome, Lobar Collapse, and Pulmonary Artery Compression: A Case Report

**DOI:** 10.7759/cureus.84993

**Published:** 2025-05-28

**Authors:** Justin D Swaby, Natalie Shahbazi, Sheryl Ang

**Affiliations:** 1 Anesthesiology, Augusta University Medical College of Georgia, Augusta, USA; 2 Radiology, Augusta University Medical College of Georgia, Augusta, USA

**Keywords:** airway edema, jet ventilation, mediastinal mass, rigid bronchoscopy, superior vena cava syndrome

## Abstract

Mediastinal masses present significant anesthetic challenges due to potential airway collapse and cardiovascular instability. We describe a case of a 68-year-old man with a locally advanced right hilar mass with extension into the mediastinum complicated by superior vena cava (SVC) syndrome, right pulmonary artery obliteration, and right upper lobe collapse who underwent rigid bronchoscopy and biopsy after failed stenting of the SVC. Careful anesthetic planning included a multidisciplinary discussion, maintenance of spontaneous ventilation, and a standby extracorporeal membrane oxygenation team. Despite initial stability, the patient experienced hypoxemic bradycardia following paralysis, requiring immediate jet ventilation via rigid bronchoscopy. Post-extubation, airway edema led to severe respiratory acidosis and necessitated reintubation. This case highlights essential principles in the anesthetic management of mediastinal masses, including patient-specific risk stratification, preparation for emergent airway interventions, and having a back-up plan in place. A structured and comprehensive perioperative plan is critical to optimize outcomes in these high-risk patients.

## Introduction

Mediastinal syndromes refer to a group of conditions characterized by the infiltration, entrapment, or compression of mediastinal structures. They are most commonly caused by malignancies such as lung cancer, with superior vena cava (SVC) syndrome being one of the most serious complications. Before induction, SVC compression impairs venous return, particularly in the supine position, leading to elevated central venous pressure and reduced preload. Induction of general anesthesia and positive pressure ventilation further decrease venous return by eliminating spontaneous respiration and muscle tone. One of the most feared complications in this setting is mediastinal mass syndrome (MMS), which occurs when a preexisting mediastinal mass causes acute airway or cardiovascular collapse during anesthesia induction due to compression of the tracheobronchial tree or great vessels. Paralysis also increases the risk of airway collapse due to loss of airway tone in the setting of tracheobronchial compression. A study of mediastinal masses reported intraoperative and postoperative complication rates of 3.8% and 10.5%, respectively [1], while the rate of major and minor complications of diagnostic bronchoscopy for patients with SVC syndrome is 6.3% and 12.5%, respectively [2]. No guidelines currently exist for the management of these patients; however, the general approach involves risk stratification followed by a risk-appropriate anesthetic plan with back-up equipment.

We present a patient with a right hilar mass extending to the mediastinum, compressing the SVC, obliterating the right pulmonary artery, and obstructing the right upper lobe bronchus who was scheduled for a rigid bronchoscopy, biopsy, and stenting. This case posed obvious perioperative concerns with regard to adequate oxygenation and ventilation as well as hemodynamic stability.

## Case presentation

A 68-year-old obese white male patient (body mass index of 34 kg/m²) with a 50-pack-year smoking history presented to the emergency room with progressive anterior neck swelling, voice changes, and orthopnea that developed over three weeks. A chest computed tomography (CT) scan revealed a right hilar mass with direct extension into the mediastinum; the mass encased and nearly obliterated the SVC and right pulmonary artery and occluded the right upper lobe bronchus. The patient underwent balloon angioplasty of the brachiocephalic vein-SVC junction by vascular surgery under conscious sedation; however, due to involvement of the cavoatrial junction and the possibility of stent migration, they opted not to place a stent. He was started on inpatient radiation therapy and scheduled for a rigid bronchoscopy with biopsy by interventional pulmonology to obtain a histopathological diagnosis for a targeted treatment plan.

**Figure 1:**
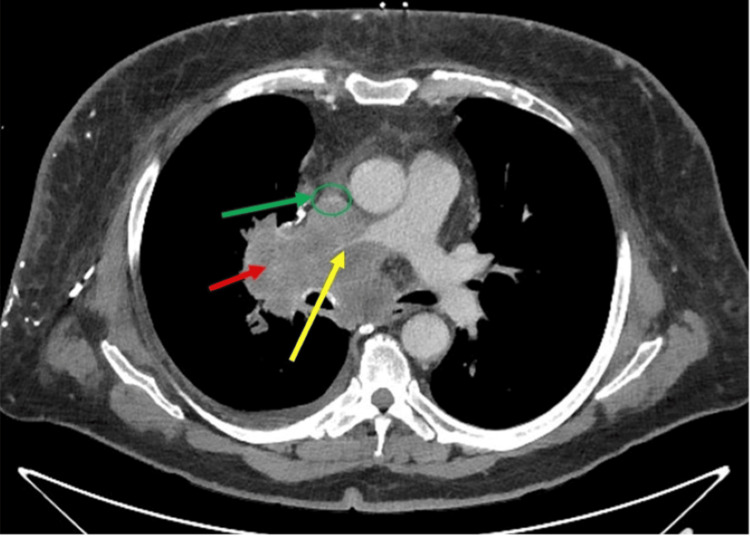
Chest CT scan with contrast (axial section) showing compression of the superior vena cava and right pulmonary artery The mediastinal mass (red arrow) is shown, encasing the superior vena cava (green circle) and right pulmonary artery (yellow arrow)

**Figure 2:**
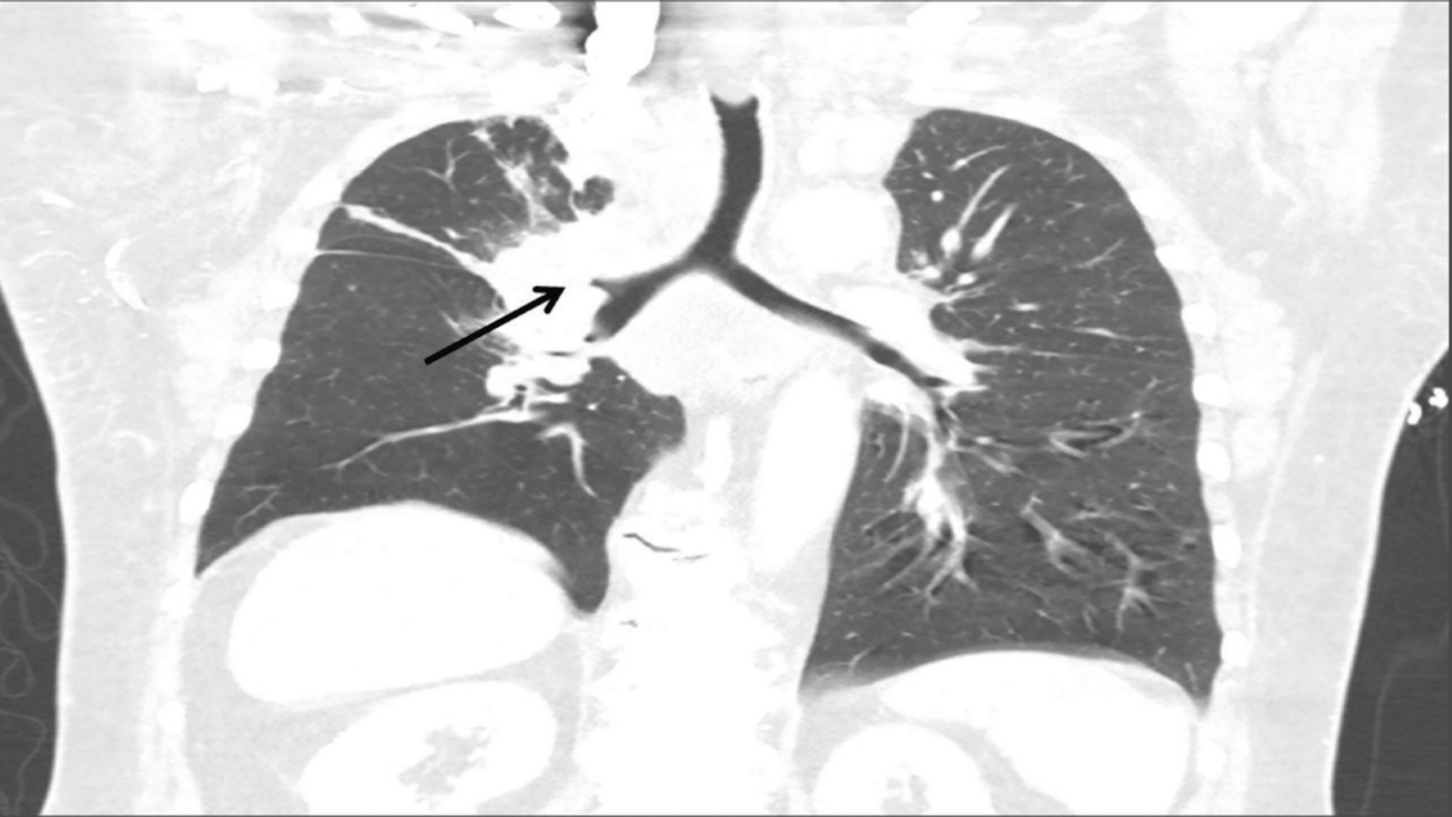
Chest CT scan (coronal view) showing compression of the right upper lobe bronchus

Preoperative evaluation of the patient revealed that he was hemodynamically stable with an oxygen saturation of 99% on 2 liters per minute (LPM) of oxygen via nasal cannula when lying supine. His physical exam was remarkable for upper body and facial swelling. Apart from Class 1 obesity, he had additional risk factors for difficult mask ventilation, such as obstructive sleep apnea and facial hair. Given the significant risk of airway and hemodynamic compromise under general anesthesia and paralysis, which were necessary for the procedure, anesthetic planning involved a multidisciplinary discussion with cardiothoracic surgery, interventional pulmonology, and perfusionists. Arrangements were made to have the cardiothoracic surgeon and perfusionist on standby for emergent extracorporeal membrane oxygenation (ECMO) in the event of difficulty in oxygenating or severe hemodynamic instability post-induction. A pre-induction arterial line was placed, and intravenous (IV) access was secured in the upper and lower extremities. Equipment for the rigid bronchoscopy and jet ventilation was ensured to be present and functioning. The patient was counseled on the high-risk nature of the procedure.

High-flow nasal cannula at 100% fraction of inspired oxygen (FiO2) and 60 LPM was used for preoxygenation. A slow and controlled intravenous induction was done with a combination of propofol infusion at 100 mcg/kg/min and remifentanil infusion at 0.05 mcg/kg/min administered via a lower extremity IV cannula while maintaining spontaneous ventilation. Hemodynamics remained stable during the induction phase. Once the patient was ascertained to be under general anesthesia, he was paralyzed with succinylcholine. Throughout this process, close communication was maintained between the anesthesia and interventional pulmonology teams. Shortly after paralysis, despite bag-valve-mask ventilation, the patient desaturated to 86% and developed profound bradycardia with a heart rate down to the low 20s. Jet ventilation via rigid bronchoscope was rapidly initiated, resulting in improvement in oxygen saturation and spontaneous normalization of his heart rate without pharmacologic intervention. Once stability in oxygenation and hemodynamics was achieved, the patient was further paralyzed with rocuronium 30mg for the procedure. During the procedure, oxygenation was maintained via jet ventilation. There was mild hypotension requiring a low-dose phenylephrine drip up to 0.5 mcg/kg/min. Fluids were administered in a controlled manner to avoid airway edema in the setting of SVC syndrome.

Following a successful bronchoscopic biopsy with tumor debulking, the pulmonology team placed an endotracheal tube for mechanical ventilation while awaiting the patient to awaken from general anesthesia. The patient was extubated awake after full reversal of the muscle relaxant and evidence of adequate minute ventilation. He was weaned off vasopressors by that time. However, immediately after extubation, he developed stridor and airway obstruction, which did not improve with positive pressure ventilation with bilateral nasal trumpets in place. There was difficulty maintaining his oxygenation saturation above 90% despite bag-valve-mask ventilation with FiO2 of 100% and bilateral nasal trumpets. He was also becoming increasingly obtunded. A stat arterial blood gas analysis revealed severe decompensated respiratory acidosis with a pH of 7.18 and a partial pressure of carbon dioxide of 78 mmHg. The decision was made to reintubate him for airway protection and mixed respiratory failure.

An inhalational induction with sevoflurane was performed while maintaining spontaneous respirations, and the patient was reintubated with the use of a video laryngoscope and a 7.0 mm internal diameter reinforced endotracheal tube. He was transferred to the medical intensive care unit for mechanical ventilatory support. His stridor and hypercarbic respiratory failure were thought to have contributed to airway edema secondary to the bronchoscopy, and he was kept intubated overnight to allow the airway swelling to subside. He was extubated uneventfully the next day to a high-flow nasal cannula after achieving optimal weaning parameters. The histopathology of the biopsy resulted in small cell carcinoma. He was started on inpatient chemotherapy with carboplatin and etoposide and subsequently discharged one week after the procedure.

## Discussion

Preoperative planning for this patient undergoing a high-risk procedure was essential for a successful perioperative outcome. The most important aim of preoperative investigation is to visualize the mass in relation to the tracheobronchial tree and vital vascular structures to determine the risk of compressive effects [3]. Although proposed radiologic and symptomatic scoring tools exist to guide the use of ECMO or cardiopulmonary bypass in patients with SVC syndrome, none have been fully validated [4]. If the mass poses risk for carinal or mainstem bronchial occlusion, bilateral vascular compression, or possible complete V/Q mismatch, preoperative ECMO should be considered [5]. Standby ECMO is often futile since the time to cannulate is roughly 5-20 minutes [6].

The traditional recommendation for patients with mediastinal masses has been to maintain spontaneous ventilation; however, newer evidence shows that positive pressure ventilation is superior, as it widens the compressed airway and increases peak inspiratory flow [7]. However, consideration should also be given to increase expiratory time during positive pressure ventilation to avoid air-trapping. Desaturation upon paralysis can be due to low functional residual capacity (FRC), airway collapse, or great vessel compression. In our patient, the improvement in saturations with jet ventilation suggests low FRC as the primary cause. Our patient's bradycardia likely resulted from a vagal response to hypoxemia, which was rectified expediently with the initiation of jet ventilation and achieving adequate oxygen saturation. The use of atropine or glycopyrrolate in this case is debatable, as it can lead to an increase in oxygen consumption and a decrease in reserves, potentially worsening hypoxia and brain injury [8].

Our patient’s airway obstruction following extubation was likely due to post-procedural laryngeal edema, necessitating reintubation. The gross incidence of post-extubation laryngeal edema ranges widely across studies, but almost 10.5% of patients with laryngeal edema will require reintubation [9]. This emphasizes the need to anticipate laryngeal edema as a potential post-procedural complication and manage it in a timely manner. Steroid prophylaxis using methylprednisolone, either 20 mg IV every 4 hours over 12 hours or a single 40 mg IV dose at least 4 hours before extubation, based on positive randomized trials, could reduce the risk of post-extubation airway complications. Notably, noninvasive ventilation does not reliably prevent reintubation in these cases, and neither heliox nor nebulized epinephrine has proven benefit in adults, although some benefits have been observed in children [10].

## Conclusions

Perioperative management of the patient with a mediastinal mass requires meticulous preoperative assessment, multidisciplinary planning, and intraoperative vigilance. While having an ECMO team on standby offers some reassurance, planned cannulation when high-risk features are present should be strongly considered. Hypoxemic bradycardia upon paralysis should prompt immediate correction of oxygenation rather than routine use of vagolytics. Finally, clinicians must anticipate post-extubation airway compromise and consider preemptive steroid administration. This case reinforces the importance of risk stratification for adequate perioperative preparation and discusses the treatment of common challenges in patients with mediastinal masses.

## References

[1] Béchard P, Létourneau L, Lacasse Y, Côté D, Bussières JS (2004). Perioperative cardiorespiratory complications in adults with mediastinal mass: incidence and risk factors. Anesthesiology.

[2] Boily-Daoust C, Plante A, Adam C, Fortin M (2021). Performance and safety of diagnostic procedures in superior vena cava syndrome. ERJ Open Res.

[3] Ku CM (2011). Anesthesia for patients with mediastinal masses. Principles and Practice of Anesthesia for Thoracic Surgery.

[4] Potere B, Boulos R, Awad H (2022). The role of extracorporeal membrane oxygenation in the anesthetic management of superior vena cava syndrome: is it time to use a scoring system?. J Cardiothorac Vasc Anesth.

[5] Saffarzadeh A, Popescu WM, Detterbeck FC, Li AX, Blasberg JD (2025). Anesthetic risk with large mediastinal masses: a management framework based on a systematic review. Ann Thorac Surg.

[6] Anderson DM, Dimitrova GT, Awad H (2011). Patient with posterior mediastinal mass requiring urgent cardiopulmonary bypass. Anesthesiology.

[7] Sarkiss M, Jimenez CA (2023). The evolution of anesthesia management of patients with anterior mediastinal mass. Mediastinum.

[8] Higgs A, Littley N, Chrimes N (2019). Bradycardia during hypoxaemic airway crises. Does atropine treat the patient or the anaesthetist?. Anaesthesia.

[9] Ak AK, Cascella M (2023). Post-intubation laryngeal edema. StatPearls.

[10] Feng IJ, Lin JW, Lai CC (2023). Comparative efficacies of various corticosteroids for preventing postextubation stridor and reintubation: a systematic review and network meta-analysis. Front Med (Lausanne).

